# Urinary arsenic species and birth outcomes in Tacna, Peru, 2019: a prospective cohort study

**DOI:** 10.14324/111.444/ucloe.3146

**Published:** 2024-12-06

**Authors:** Diego Fano-Sizgorich, Matthew O. Gribble, Cinthya Vásquez-Velásquez, Claudio Ramírez-Atencio, Julio Aguilar, Jeffrey K. Wickliffe, Maureen Y. Lichtveld, Dana B. Barr, Gustavo F. Gonzales

**Affiliations:** 1 Laboratorio de Endocrinología y Reproducción, Laboratorios de Investigación y Desarrollo (LID), Facultad de Ciencias e Ingeniería, Universidad Peruana Cayetano Heredia, Lima, Peru; 2 Department of Medicine, Division of Occupational, Environmental, and Climate Medicine, University of California, San Francisco, San Francisco, CA, USA; 3 Facultad de Ciencias de la Salud, Universidad Nacional Jorge Basadre Grohmann, Tacna, Peru; 4 Department of Environmental Health Sciences, School of Public Health, University of Alabama at Birmingham, Birmingham, AL, USA; 5 School of Public Health, University of Pittsburgh, Pittsburgh, PA, USA; 6 Gangarosa Department of Environmental Health, Rollins School of Public Health, Emory University, Atlanta, GA, USA

**Keywords:** birth weight, foetal development, gestational age, toxicity, pregnant women, Latin America

## Abstract

Arsenic exposure during pregnancy might affect foetal development. Arsenic metabolism may modulate the potential damage to the fetus. Tacna has the highest arsenic exposure levels in Peru. However, this region also has the highest birth weight in Peru. It is not known if arsenic exposure is affecting maternal–perinatal health in Tacna. This study aimed to evaluate the association between urinary arsenic metabolism and birth outcomes, specifically birth weight and gestational age at birth in Tacna, Peru. A prospective cohort study was conducted, involving 158 pregnant women in Tacna, Peru, during January–November 2019. Participants were enrolled in their second trimester and followed-up until birth. Urine samples were collected in the second and third trimesters. Urine samples were analysed for total arsenic concentration and its species. Generalised estimating equations analysis was used to evaluate the association of interest. Inter-differences in arsenic toxicokinetics, calculated with principal component analysis was included as an interaction term. Analysis was stratified by pregnancy trimester. The median total urinary arsenic concentration was 33.34 μg/L. Inorganic arsenic and dimethylarsinic acid were higher in the second trimester. Dimethylarsinic acid was the predominant component (84.78% of total urinary arsenic). No significant association was found between urinary arsenic exposure and birth weight or gestational age at birth. The association was not affected by arsenic metabolism. Stratified analyses by pregnancy trimester also showed no significant associations. Urinary arsenic was not associated with birth weight, and this null relationship remained unaffected by arsenic toxicokinetic differences reflected in urine.

## Introduction

Arsenic is a naturally occurring element found in the Earth’s crust, soil, water and air. It is a toxic substance and a known carcinogen, causing skin, lung, bladder and kidney cancers [1]. Arsenic is also known to have adverse effects on foetal and infant health [2]. Pregnant women who are exposed to high levels of arsenic are at an increased risk of adverse birth outcomes, including stillbirth, preterm birth (<37 weeks of gestational age), low birth weight (<2500 g at term) and congenital abnormalities [3]. In recent years, there has been growing concern about the impact of arsenic exposure on maternal and child health.

The ingestion of water containing high concentration of arsenic is one of the most common routes of exposure. It is estimated that 107 countries around the world are affected by high levels of arsenic in water [4], with groundwater being the most common source, although high levels are also found in surface water [5]. Arsenic concentration in water can be very heterogeneous even in a same country, such as Bangladesh, with arsenic levels ranging from 90 to 4730 μg/L in tube-well water [6]. In Chile, at Bahía de Camarones, which is located near the city of Arica (border with Peru), drinking water inorganic arsenic (iAs) levels of 48.7–1252 μg/L have been found, composed particularly of arsenate (As^V^) [7]. A study from our group has determined that around two-thirds of the Tacna (a province in southern Peru) population of pregnant women are exposed to iAs levels higher than 10 μg/L in tap water, of which 50% were exposed to >50 μ/L [8]. However, Tacna, despite the arsenic exposure context, has shown the highest birth weight in Peru [9,10], as well as the lowest small-for-gestational age prevalence [10].

Urinary arsenic and its metabolites are commonly used as biomarkers of arsenic exposure in epidemiological studies [11]. Arsenic and its metabolites are excreted primarily in urine, and urinary arsenic levels have been shown to correlate with the internal dose of arsenic exposure [11]. Several studies have reported a significant association between maternal urinary arsenic levels and adverse birth outcomes, although the findings have been inconsistent across studies [3,12]. It is important to note that individuals have varying proficiencies in metabolising arsenic, and this could modulate the potential damage to the fetus [13].

Given the potential health risks associated with arsenic exposure during pregnancy, there is a need for further research to better understand the impact of arsenic on maternal and child health. This study aims to evaluate the association between urinary arsenic metabolism and birth outcomes, specifically birth weight and gestational age at birth.

## Materials and methods

### Study design and study area

We conducted a longitudinal cohort study during January–November 2019, in which a total of 158 pregnant women living in the province of Tacna, in their second trimester of pregnancy who attended their antenatal care-controls were enrolled and followed-up until birth. The province of Tacna is in southern Peru, with a total area of 8170 km^2^, and it is characterised for its desertic geography.

### Enrolment of participants and follow-up

The recruitment of the pregnant women is described elsewhere [8]. In brief, a total of 16 health establishments within the five most populated districts in the province of Tacna were selected for the enrolment to take place. We were granted authorisation to consult the prenatal health care record, which included information about the date of last antenatal care consultation, gestational age by the time of consultation, age, address and telephone number.

To be considered as a potential participant for the study, the women were 18–40 years old, had lived in Tacna for at least 5 years, and were pregnant for <24 weeks by the time of the recruitment. Eligible women were recruited via a telephone call. Those invited to participate in the study were then visited in their homes or in the health establishment a total of two times for urine sampling. A final visit was scheduled after birth, in which data from their baby was collected, such as birth weight and gestational age at birth.

### Urine sampling and arsenic quantification

One urine sample was taken in the second and third trimester of pregnancy. During the recruitment the women were given two sterile plastic flasks for urine specimen collection. They were asked to avoid consuming fish or seafood for the last three days prior the sampling. They were instructed in how to do the self-collection of the sample, indicating that they should eliminate the first few millilitres of the morning void. Once the sample was collected, participants were asked to store it in the freezer until the research personnel were able to collect them. The samples were transported at 4°C to the laboratory for storage. Samples were homogenised and then aliquoted in cryovials of 2 mL, and stored at −20°C. For arsenic quantification and speciation, the samples were delivered on dry ice to the LEADER laboratory at Emory University in Atlanta, GA, USA. The procedure is described elsewhere [14].

### Statistical analysis

Descriptive statistics were used to display median with interquartile range for non-normal distributed data. Categorical variables are presented as absolute and relative frequencies. Arsenic species concentrations and their relative per cent (%) are presented.

Relative percent of the species were calculated as follows:

$$\text{\%iAs}=\frac{\left[{\text{As}}^{\text{III}}]+[{\text{As}}^{\text{V}}\right]}{[{\text{As}}^{\text{III}}]+[{\text{As}}^{\text{V}}]+[\text{MMA}]+\text{[DMA]}},$$

$$\text{\%MMA}=\frac{\left[\text{MMA}\right]}{[{\text{As}}^{\text{III}}]+[{\text{As}}^{\text{V}}]+[\text{MMA}]+\text{[DMA]}},$$

$$\text{\%DMA}=\frac{\left[\text{DMA}\right]}{[{\text{As}}^{\text{III}}]+[{\text{As}}^{\text{V}}]+[\text{MMA}]+\text{[DMA]}},$$

where

[iAs]: inorganic arsenic concentration in urine

[As^III^]: arsenate concentration in urine

[As^V^]: arsenate concentration in urine

[MMA]: monomethylarsonic acid concentration in urine

[DMA]: dimethylarsinic acid concentration in urine

**Figure A1:**
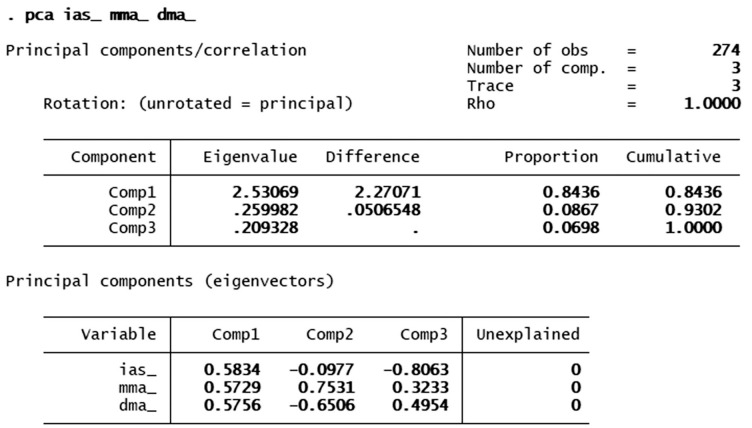
Arsenic principal component analysis, correlation and eigenvectors.

To compare total urinary arsenic (tAs) and arsenic species concentration between the second and third trimester of pregnancy, we used Wilcoxon’s signed-rank test. We used Student’s t-test for paired observations to compare if %iAs, %MMA and %DMA were different between pregnancy trimesters, after the normal distribution evaluation of the differences. We performed a principal component analysis (PCA) to characterise the main sources of variability in the urinary arsenic data and its species (arsenic toxicokinetics differences between pregnant women). The PCA was conducted on the concentration of urinary iAs, MMA and DMA. The principal components correlations and eigenvectors can be found in Fig. A1.

Arsenic exposure was considered as the residuals of the following model to remove the influence of organic arsenic from seafood on urinary total arsenic [15,16]:

$$\text{tAs}=\beta_{\text{1}}*\text{Asb}+\beta_{\text{2}}*{\text{Asb}}^{\text{2}}+\text{constant,}$$

where

tAs: total urinary arsenic (μg/L)

Asb: arsenobetaine (μg/L)

Generalised estimating equations (GEE) with Gaussian family analysis was employed to evaluate the association between arsenic and birth weight, and whether this association was affected by arsenic toxicokinetic differences between pregnant women. This same approach was applied to examine the association with gestational age at birth, but as coefficients were small, the variable arsenic exposure was scaled by dividing it by 1000 for better interpretation. GEE analysis was then stratified by newborn sex. An analysis stratified by pregnancy trimester was performed using linear regression. Regression models were adjusted for mother’s age, pregestational body mass index (BMI) and mother’s education level (as a proxy for socioeconomic status). All statistical analyses were conducted using STATA 17.0 software with a significance level of *p* < 0.05.

## Results

The study sample of pregnant women had a mean age of 28.15 years at the time of recruitment, and mean BMI of 26.73 kg/m^2^ before pregnancy. Only five women (3.13%) declared to be smokers during pregnancy and 13 consumed alcohol (8.16%). Thirty-six of the women (22.50) were single mothers, and the sample had a high proportion of women with higher education (38.13%). In Table 1 we present the distribution of urinary arsenic species concentrations as median and interquartile range (IQR). Median total tAs was 33.34 μg/L and ranged between 2.50 and 167.48 μg/L. We observed variation in tAs across visits, being lower in visit 2. DMA was the most present arsenic component (84.78%). Water arsenic concentration distribution in the second and third trimester can be found in Table A1, indicating that for the third trimester, pregnant women were mostly exposed to levels ≤10 μg/L (51.83% vs. 29.56% in the second trimester), and there was a positive significant correlation between water arsenic and urinary DMA concentration in both trimesters.

**Table 1.** Urinary arsenic species concentration and relative content across pregnancy

Arsenic species (μg/L)	Total		Second trimester		Third trimester		*p*-value^a^
	Median	IQR	Median	IQR	Median	IQR	
tAs	33.34	30.58	41.57	33.95	28.32	20.67	<0.001
AsIII	1.57	1.57	2.08	1.90	1.24	1.03	<0.001
AsV	1.36	1.30	1.36	1.36	1.36	1.21	0.553
iAs	2.99	2.80	3.54	2.99	2.68	2.03	0.001
MMA	2.10	1.79	2.17	2.07	2.07	1.35	0.165
DMA	28.36	26.86	35.55	29.06	23.36	16.75	<0.001
Asb	2.37	2.55	2.64	3.05	2.09	2.24	0.002
%iAs^b^	8.85	2.72	8.30	2.59	9.49	2.73	<0.001
%MMA^b^	6.37	2.21	5.41	1.87	7.47	2.06	<0.001
%DMA^b^	84.78	4.05	86.28	3.56	83.03	3.89	<0.001

^a^Wilcoxon’s signed-rank for total arsenic and arsenic species concentration; and paired Student’s t-test for arsenic species relative content (%).

^b^Values are shown as mean and standard deviation.

Mean birth weight was 3618 ± 477.38 g. As seen in Table 2, there was no significant association between urinary arsenic and birth weight [adjusted β = 0.16, 95% confidence interval (CI) −1.07; 1.39, *p* = 0.800]. The interaction between urinary arsenic and arsenic toxicokinetics difference between women (PCA Score 1) showed a reduction in birth weight; nonetheless, this was non-significant (adjusted β = −0.05, 95% CI −0.76; 0.65, *p* = 0.882).

**Table 2.** Association between urinary arsenic and interaction with arsenic metabolism with birth weight

Variable	Unadjusted	95% CI	Adjusted	95% CI
Urinary arsenic	0.04	−1.27; 1.36	0.16	−1.07; 1.39
Score 1^a^	0.62	−16.09; 17.33	1.27	−14.11; 16.65
Urinary arsenic^a^ Score 1	−0.10	−0.89; 0.69	−0.05	−0.76; 0.65
Mother’s age	4.40	−7.81; 16.60	3.63	−7.97; 15.23
Pregestational BMI	**23.76**	**9.59; 37.92**	**20.65**	**6.94; 34.35**
Education				
Elementary	Ref.		Ref.	
Secondary	305.65	−1.68; 612.97	**371.28**	**72.67; 669.93**
Tertiary	212.99	−101.37; 527.36	**312.73**	**8.70; 616.77**

Residuals were calculated from the model tAs~β_1_ (Asb) + β_2_ (Asb)^2^.

Models were adjusted for mother’s age, mother’s education level, pregestational BMI.

^a^Score 1 (arsenic toxicokinetics difference between women), obtained from principal components analysis, is higher when %DMA is lower, meaning a reduced metabolic capability.

**Bold** letters indicate a *p* < 0.05.

Regarding gestational age at birth, as seen in Table 3, we found a non-significant increase of 0.02 weeks (95% CI −2.37; 2.40, *p* = 0.989), while the interaction term presented a decrease, although not significant, in gestational age at birth (β = −0.17, 95% CI −1.53; 1.19, *p* = 0.802).

**Table 3.** Association between urinary arsenic and interaction with arsenic metabolism with gestational age at birth

Variable	Adjusted	95% CI	Adjusted	95% CI
Urinary arsenic	−0.08	−2.48; 2.32	0.02	−2.37; 2.40
Score 1^a^	0.01	−0.02; 0.04	0.01	−0.02; 0.04
Urinary arsenic^a^ Score 1	−0.19	−1.63; 1.24	−0.17	−1.53; 1.19
Mother’s age	**−0.03**	**−0.06; −0.004**	**−0.03**	**−0.06; −0.001**
Pregestational BMI	**−0.04**	**−0.07; −0.01**	−0.03	−0.07; 0.003
Education				
Elementary	Ref.		Ref.	
Secondary	0.62	−0.12; 1.36	0.48	−0.27; 1.23
Tertiary	0.55	−0.21; 1.31	0.32	−0.45; 1.08

Residuals were calculated from the model tAs~β_1_ (Asb) + β_2_ (Asb)^2^.

Models were adjusted for mother’s age, mother’s education level, pregestational BMI.

^a^Score 1 (arsenic toxicokinetics difference between women), obtained from principal components analysis, is higher when %DMA is lower, meaning a reduced metabolic capability.

**Bold** letters indicate a *p* < 0.05.

In the stratified analysis by newborn sex, no significant association was found between arsenic exposure or the interaction term related to arsenic toxicokinetic differences and birth weight. However, for gestational age at birth, a significant association (*p* = 0.041) was observed for males, indicating that each increase of 1000 units in urinary arsenic exposure is associated with an increase of 7.36 weeks in gestational age at birth (Table 4).

**Table 4.** Association between urinary arsenic and interaction with arsenic metabolism with birth weight and gestational age at birth stratified by newborn sex

Newborn sex	Regression term	Birth weight		Gestational age at birth	
		Adjusted	*p*-value	Adjusted	*p*-value
Male	Urinary arsenic	2.79 (−0.02; 5.60)	0.052	7.36 (0.30; 14.42)	0.041
	Urinary arsenic^a^ Score 1^a^	0.36 (−1.41; 2.13)	0.689	−2.79 (−8.84; 3.26)	0.364
Female	Urinary arsenic	−0.47 (−4.27; 3.32)	0.806	−6.92 (−16.62; 2.77)	0.160
	Urinary arsenic^a^ Score 1	−1.41 (−3.79; 0.98)	0.245	0.95 (−4.83; 6.72)	0.745

Regressions were adjusted for mother’s age, pregestational BMI and education.

Coefficients for gestational age at birth are scaled (urinary arsenic/1000).

^a^PCA Score 1 (arsenic toxicokinetics difference between women) is higher when %DMA is lower, meaning a reduced metabolic capability.

For both models, the adjusted regression coefficient (95% CI) is shown.

We then evaluated if arsenic or the interaction term with arsenic toxicokinetic differences were associated with both outcomes, stratifying it by pregnancy trimester. As seen in Table 5, there was no association between urinary arsenic exposure and the interaction term with birth weight and gestational age at birth.

**Table 5.** Association between urinary arsenic and interaction with arsenic metabolism with birth weight and gestational age at birth stratified by visit

Trimester	Regression term	Birth weight		Gestational age at birth	
		Adjusted	*p*-value	Adjusted	*p*-value
Second	Urinary arsenic	1.61 (−1.44; 4.67)	0.298	−5.11 (−14.43; 4.20)	0.280
	Urinary arsenic^a^ Score 1^a^	−1.36 (−3.32; 0.59)	0.170	−5.13 (−12.00; 1.75)	0.142
Third	Urinary arsenic	−1.91 (−6.09; 2.27)	0.368	7.88 (−5.81; 21.57)	0.257
	Urinary arsenic^a^ Score 1	1.60 (−0.84; 4.05)	0.197	−0.81 (−8.19; 6.57)	0.828

Regressions were adjusted for mother’s age, pregestational BMI and education.

Coefficients for gestational age at birth are scaled (urinary arsenic/1000).

^a^PCA Score 1 (arsenic toxicokinetics difference between women) is higher when %DMA is lower, meaning a reduced metabolic capability.

For both models, the adjusted regression coefficient (95% CI) is shown.

## Discussion

The present study aimed to evaluate the association between urinary arsenic and metabolism with birth weight and gestational age at birth. No association with these outcomes was found, and this null relationship is unaffected by arsenic toxicokinetic differences reflected in urine.

No association may have been found because exposure levels might not be high enough to exert an effect. Previous studies have found a decrease in birth weight with increasing levels of urinary arsenic, at exposure levels ≥100 μg/L.^3^ In this study, the median level of urinary arsenic for the cohort across pregnancy was 33.34 μg/L with a range of 2.50–167.48 μg/L. A total of 25 and 36 women showed urinary tAs levels ≥100 μg/L in the second and third trimester of pregnancy, respectively, but no difference in birth weight was found (Table A2). In some previous studies, low levels of arsenic in urine (1.8–27.7 μg/L) have not been found to be associated with a decrease in birth weight [17]. However, other studies with similar exposure levels in urine have found a significant association with birth weight or estimated foetal weight [18,19]. A Wuhan cohort study that showed median urinary arsenic levels of 31.22 μg/L for the first, 25.23 μg/L for the second and 24.98 μg/L for the third trimester found a significant decrease of 24.27 g in birth weight only for the third trimester [12]. This suggests that even low exposure levels might be harmful for foetal development. Additionally, it is important to remark that no arsenic exposure level is considered to be safe as even water arsenic exposure levels between 1 and 10 μg/L have been associated with increased cardiovascular mortality compared to concentrations <1 μg/L [20].

In a cohort study from Bangladesh, it was found that water and toenail arsenic association with birth weight was mediated by gestational age [21,22]. In the present study, pregnancy duration, seen as gestational age at birth, was not associated with arsenic exposure. This difference might be attributed to the level of arsenic exposure in drinking water observed in the Bangladeshi cohort. Although the median arsenic concentration was 2.3 μg/L at the time of enrolment, 33.3% of pregnant women were exposed to levels ranging from 18.4 to 1400 μg/L [21]. On the one hand, it has been found that low arsenic levels in biological samples such as umbilical cord (3.82 ± 3.81 μg/L) and whole blood (4.13 ± 3.21 μg/L) were associated with a decrease in gestational age by 0.342 weeks [23]. On the other hand, in a study that included a total of 212 mother–infant pairs, no association was found between total urinary arsenic (median 7.77 μg/L) and urinary DMA (3.44 μg/L) with gestational age [24]. The lack of association with birth weight and gestational age at birth could be due to an exposure below harmful levels, or to unmeasured nutritional, genetical and other factors.

When analysing the impact of arsenic exposure on birth outcomes by newborn sex, we found no significant relationship between arsenic levels and birth weight. However, for male infants, there was a notable increase in gestational age – specifically, an increase of 0.0746 weeks for every 10 units rise in urinary arsenic concentration. In contrast, a previous study involving 113 mother–child pairs reported no significant associations between arsenic exposure and gestational age across both sexes [25]. This discrepancy may stem from different exposure levels, particularly if Tacna has higher arsenic concentrations. Despite the modest effect size observed in our study, it remains unclear why urinary arsenic correlates positively with gestational age.

Arsenic can be metabolised, and a higher arsenic methylation capability of the body can reduce this metalloid toxicity [26]. Higher concentration of urinary MMA and urinary iAs are shown to have the biggest impact in decreasing birth weight and birth length, respectively [13]; evidence is less clear for DMA. Nonetheless, a higher proportion of DMA, which means a better arsenic metabolism, is associated with better health outcomes compared to those with lower DMA, such as general health status of children [27] and neurodevelopment in low birth weight preterm children [28]. We have observed in pregnant women from Tacna, Peru that DMA at 84.78% (tAs minus Asb) represents the main arsenic component present in urine. This may explain the low negative impact of arsenic on birthweight and gestational age at birth; and suggests that the difference in arsenic toxicokinetics might modify the association.

The effect modification of arsenic toxicokinetics was also assessed in the study by including the interaction term of arsenic with the PCA Score 1. For both birth weight and gestational age at birth, differences in arsenic metabolism seemed to modify the association by reducing these outcomes, although it was non-significant. Despite not finding an association, there might be an interaction between arsenic exposure and metabolism, as suggested in a Romanian longitudinal pilot study, where women who had low birth weight children showed a higher percentage of iAs and MMA [29], suggesting a slower or reduced metabolism.

Consideration of arsenic species and speciation is essential for a better understanding of exposure, not only in research studies but also in nationwide screenings such as the one done in the National Health and Nutrition Examination Survey (NHANES) [30,31]. Currently, the Peruvian Demographic and Health Survey does not consider water or urinary arsenic evaluation.

It is possible that birth weight was not affected due to the variation in arsenic exposure between pregnancy trimesters. Other studies showed that there are seasonal variations in water and urinary arsenic concentration [32–34], although depending on the area, the change can be very small (3.3 μg/L in well water between the dry and rainy season) [35]. The first study visit was conducted in the summer and autumn, while the second visit occurred during the winter and spring. At the second visit, median tAs was 28.32 μg/L, compared with 41.57 μg/L found in the first study visit. In the stratified analysis, no association was found with arsenic exposure, nor with toxicokinetic differences.

The fetus experiences the fastest weight gain during the third trimester [36], and different arsenic exposures in this developmental window have been found to reduce birth weight [37], although some authors have found that early pregnancy arsenic exposure might be the critical window for birth weight and other pregnancy outcomes [38] Nonetheless, trimester-based analysis might not reflect an adequate association [39]. Daily exposure assessment is difficult for exposures that need biological samples such as urinary arsenic. Arsenic has been found to be associated with a decrease in birth weight and gestational age at birth, possibly through lowering the thyroid hormones ratio during early pregnancy [18]. Seasonal variation in exposure, along with the analysis of pregnancy-relevant hormones, should be considered for a better evaluation and interpretation.

It is notable that pregnant women from Tacna, despite living in the highest arsenic-exposed region in Peru, have one of the highest mean birth weights [10]. One contributing factor may be the considerable proportion of individuals from the Aymara ethnicity in Tacna [8,14]. This is an indigenous group, predominantly located in high-altitude settings, that is known for higher birth weight compared to other high-altitude populations [40]. In our sample, neonates of pregnant women who self-identified as Aymara had a mean birth weight of 3711 g, higher compared to the other ethnic groups (3536 g for mestizo and 3466 g for Quechua) (Table A3). These findings suggests that the Aymara population may possess genetic traits that support foetal weight gain, even in the context of arsenic exposure.

When considering arsenic metabolism, polymorphisms in the *AS3MT* gene-related increased arsenic metabolic capability [41–44], were found in Aymara populations of Argentina [45]. However, while 55.41% of our sample self-identified as Aymara, %DMA was not different between the ethnic groups in our study (Table A4). These hypotheses should be explored in further studies.

The study has some limitations. There were unmeasured confounders such as the consumption of folates, which are part of the one-carbon metabolism and methyl donors for arsenic metabolism, which could modify the association between arsenic metabolism and birth weight [46]. Based on the Peruvian national programme on pregnancy, it is mandatory to supplement pregnant women with folic acid; therefore, the folate deficiency in our population is reduced; however, folate intake should be considered in further studies. Covariates such as gestational weight gain should also be evaluated as it is strongly associated with birth weight, especially during the first half of gestation [47]. The exposure assessment at the beginning of pregnancy (first trimester) is encouraged, as it would also allow testing of the effects of arsenic on placenta formation, as has been suggested in both human [48] and animal studies [49]. This would also allow for a better evaluation of seasonal variation in arsenic exposure. This study used specific gravity to adjust arsenic concentration in urine, which may have different sources of measurement error than, for instance, creatinine adjustment [50].

## Conclusions

Arsenic was not associated with birth weight or gestational age at birth in this study, and this null relationship was unaffected by arsenic toxicokinetic differences reflected in the analysis. This should not be interpreted as if the Tacna population is protected against arsenic toxicity. Further studies should include other variables to better understand this phenomenon and the mechanism(s) behind it, including the evaluation of other clinical outcomes. Additionally, the inclusion of arsenic exposure assessment and its speciation in national programmes should be encouraged for better monitoring, along with the elimination of arsenic contamination in drinking water.

## Data Availability

The datasets generated during and/or analysed during the current study are available from the corresponding author on reasonable request.

## Appendix

**Table A1.** Water arsenic concentrations distribution of pregnant women in the second and third trimester, and its correlation with urinary DMA

Water arsenic level category (μg/L)^a^	Second trimester			Third trimester		
	No. pregnant women	%	DMA correlation^b^	No. pregnant women	%	DMA correlation^b^
5	15	9.43	0.345**	24	17.52	0.279*
10	32	20.13		47	34.31	
25	55	34.59		39	28.47	
50	33	20.75		19	13.87	
100	22	13.84		5	3.65	
250	2	1.26		3	2.19	

^a^Water arsenic concentrations were obtained by analysing household drinking water samples, using a semi-quantitative method described in [8].

^b^Spearman’s correlation analysis (Spearman’s rho).

**p* < 0.01, ***p* < 0.001.

**Table A2.** Mean birth weight comparison between women with total urinary arsenic exposure levels ≥100 μg/L and <100 μg/L by trimester of pregnancy

Trimester	tAs exposure (μg/L)	No. participants	Birth weight		*p*-value*
			Mean	SD	
Second	<100	122	3623.82	489.99	0.749
	≥100	25	3589.79	412.38	
Third	<100	91	3622.06	430.73	0.865
	≥100	36	3606.32	631.28	

SD: Standard deviation.

**p*-value for Student’s t-test.

**Table A3.** Mean birth weight according to the mother’s self-reported ethnic group, and one-way analysis of variance (ANOVA) analysis

Ethnic group (n)	Birth weight		*p*-value*
	Mean	SD	
Mestizo (52)	3536.06	486	0.037
Quechua (18)	3466.11	391.47	
Aymara (87)	3711.38	480.2	

The group size for each ethnic group is displayed in parenthesis.

**p*-value for one-way ANOVA test.

**Table A4.** Percentage of dimethylarsinic acid (%DMA) in different ethnic groups

Ethnic group (n)	%DMA		*p*-value*
	Mean	SD	
Mestizo (43)	84.88	4.10	0.463
Quechua (14)	84.99	4.23	
Aymara (65)	84.66	4.02	

The group size for each ethnic group is displayed in parenthesis.

**p*-value for one-way ANOVA test.
